# Intravenous Iron Supplementation Practices and Short-Term Risk of Cardiovascular Events in Hemodialysis Patients

**DOI:** 10.1371/journal.pone.0078930

**Published:** 2013-11-01

**Authors:** Abhijit V. Kshirsagar, Janet K. Freburger, Alan R. Ellis, Lily Wang, Wolfgang C. Winkelmayer, M. Alan Brookhart

**Affiliations:** 1 University of North Carolina Kidney Center, UNC School of Medicine, Chapel Hill, North Carolina, United States of America; 2 Cecil G. Sheps Center for Health Services Research, University of North Carolina, Chapel Hill, North Carolina, United States of America; 3 Division of Nephrology, Department of Medicine, Stanford University School of Medicine, Palo Alto, California, United States of America; 4 Department of Epidemiology, UNC Gillings School of Global Public Health, UNC, Chapel Hill, Chapel Hill, North Carolina, United States of America; Institut national de la santé et de la recherche médicale (INSERM), France

## Abstract

**Background & Objectives:**

Intravenous iron supplementation is widespread in the hemodialysis population, but there is uncertainty about the safest dosing strategy. We compared the safety of different intravenous iron dosing practices on the risk of adverse cardiovascular outcomes in a large population of hemodialysis patients.

**Design settings, participants, & measurements:**

A retrospective cohort was created from the clinical database of a large dialysis provider (years 2004-2008) merged with administrative data from the United States Renal Data System. Dosing comparisons were (1) bolus (consecutive doses ≥ 100 mg exceeding 600 mg during one month) versus maintenance (all other iron doses during the month); and (2) high (> 200 mg over 1 month) versus low dose (≤ 200 mg over 1 month). We established a 6-month baseline period (to identify potential confounders and effect modifiers), a one-month iron exposure period, and a three-month follow-up period. Outcomes were myocardial infarction, stroke, and death from cardiovascular disease.

**Results:**

117,050 patients contributed 776,203 unique iron exposure/follow-up periods. After adjustment, we found no significant associations of bolus dose versus maintenance, hazards ratio for composite outcome, 1.03 (95% C.I. 0.99, 1.07), or high dose versus low dose intravenous iron, hazards ratio for composite outcome, 0.99 (95% C.I. 0.96, 1.03). There were no consistent associations of either high or bolus dose versus low or maintenance respectively among pre-specified subgroups.

**Conclusions:**

Strategies favoring large doses of intravenous iron were not associated with increased short-term cardiovascular morbidity and mortality. Investigation of the long-term safety of the various intravenous iron supplementation strategies may still be warranted.

## Introduction

Erythropoiesis stimulating agents (ESAs) and intravenous (IV) iron have been used in combination to manage anemia in the dialysis population for many years. Clinical trials providing new safety information about ESAs [1-3] and the implementation of capitated payments for dialysis services [4] have led to a relative shift away from ESA use. The use of IV iron for the management of anemia during the same period has grown [5].

 Yet, little is known about the safety of IV iron. In contemporary practice, intravenous iron administration varies among clinicians and dialysis units [6,7]. Some providers administer large sequential doses of approximately 1 gram per month and others, aliquots of 50-100 mg spaced over weeks. Any single patient may be exposed to either of these dosing strategies during his or her time receiving hemodialysis.

While the optimal dosing regimen for IV iron remains unknown, biological mechanisms suggest that the sub-optimal use of iron could lead to adverse clinical events [8]. Free iron is a potent oxidizing agent that can catalyze the formation of highly reactive oxygen species [9,10]. These highly reactive oxygen species could give rise to lipid radicals, which may lead to endothelial dysfunction [11] and atherogenesis [12], possibly increasing the risk of cardiovascular events in a population already known to have a high burden of cardiovascular disease [13].

We therefore conducted a large-scale retrospective observational study using data from a large national dialysis provider linked with the United States Renal Data System (USRDS). Our goal was to examine the comparative short-term cardiovascular safety of common dosing strategies of IV iron in a cohort of contemporary patients receiving hemodialysis, and among pre-specified subgroups of patients.

##  Materials and Methods

### Data Sources

The data used for this study came from the clinical research database of a large dialysis provider and the USRDS. The dialysis provider owns and manages over 1,500 outpatient dialysis facilities located throughout the United States in urban, rural, and suburban areas. Their clinical database captures detailed clinical, laboratory, and treatment data on patients receiving care at all of their dialysis units. All data are collected using standardized electronic health record systems. For this study, we used the clinical data to obtain detailed information on IV iron dosing, epoetin alfa (EPO) use and dosing, clinical laboratory values (e.g., hemoglobin, transferrin saturation (TSAT), serum ferritin), and current vascular access. The USRDS is a national data system funded by the National Institutes of Diabetes and Digestive and Kidney Diseases that collects, analyzes, and distributes information about the treatment of end stage renal disease (ESRD). The USRDS data includes data from the Medical Evidence Report Form, the Medicare Enrollment database, the ESRD Death Notification Form, and the standard analytic files, which contain final action claims data [14].

 We examined 5 years of data (2004 - 2008) from the clinical database to identify the cohort. These data were merged with data from the USRDS to obtain information on demographic characteristics, health care use (e.g., hospitalizations, outpatient care), and additional clinical characteristics (e.g., comorbidities).

### Study Design

**Figure 1:**
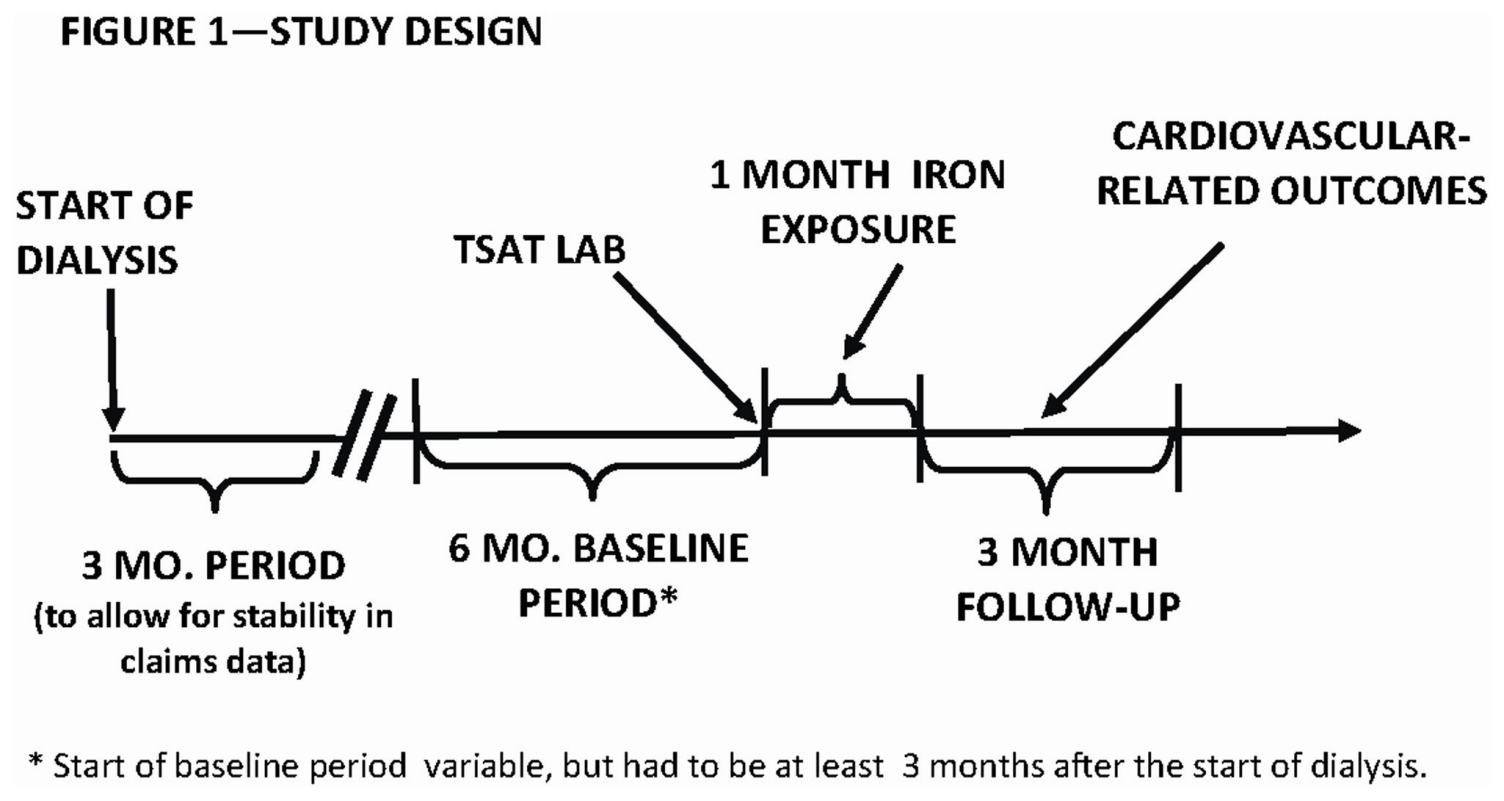
Study Design.

We utilized a retrospective cohort design in which we established a 6-month baseline period (to identify potential confounders and effect modifiers), a one-month iron exposure period, and a three-month follow-up period (see Figure 1). The index date of the exposure period was anchored on the day of a laboratory assessment of TSAT, because this information is used to guide subsequent iron administration. 

### Cohort Identification

**Figure 2:**
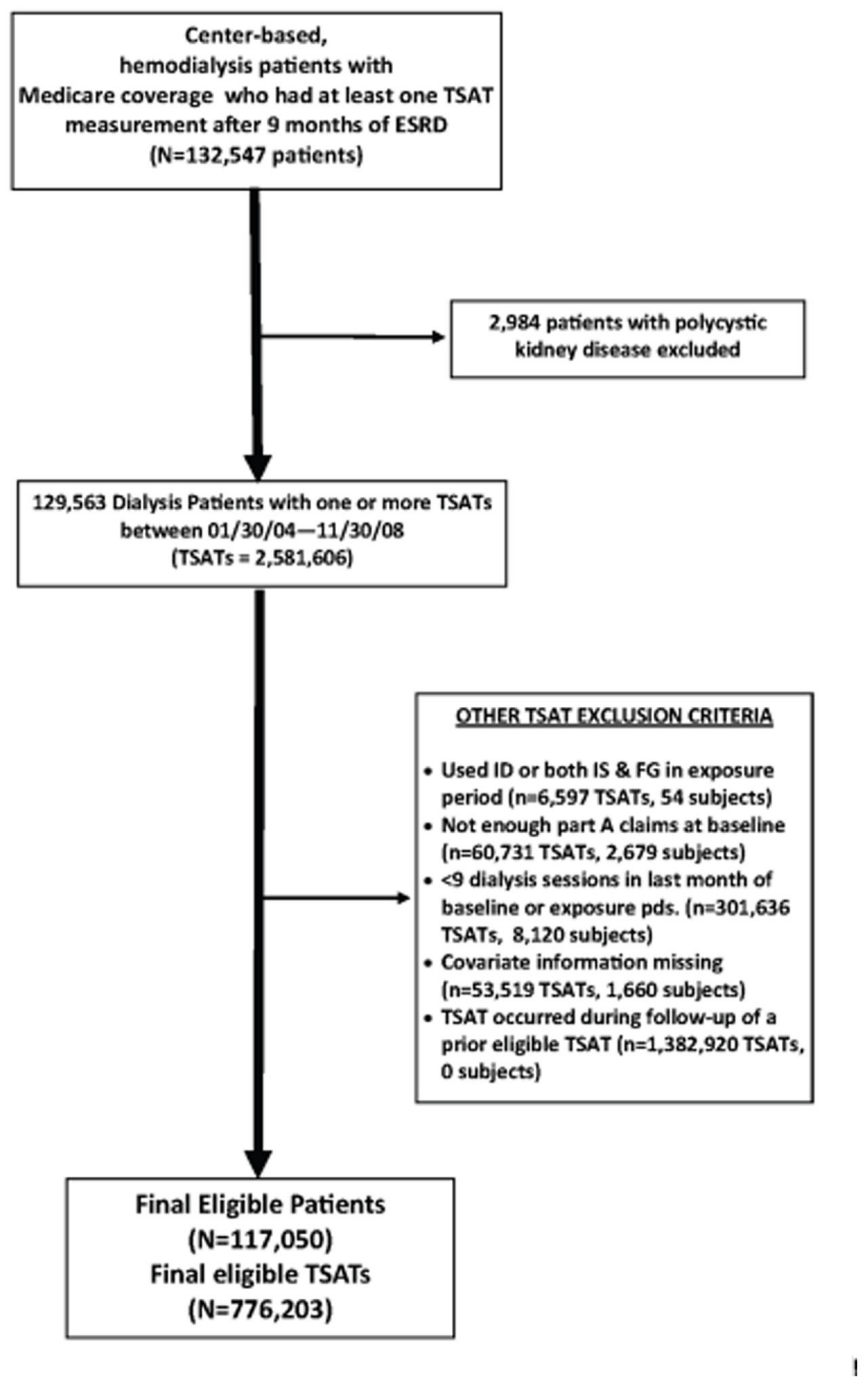
Cohort Identification.

Figure 2 depicts the creation of our sample. Following the merge of the clinical and USRDS data, we first identified center-based, hemodialysis patients who had at least one TSAT measurement after undergoing dialysis for at least 9 months. The 9-month period accounted for the 6-month baseline period and an additional 3 months from the start of dialysis to allow for stability in the claims processing by the Centers for Medicare and Medicaid Services (CMS) [14]. Individuals with polycystic kidney disease were then excluded because of the low use of ESA’s or IV iron. Eligible patients could contribute more than one TSAT measurement. TSAT measurements were eligible if they occurred between January 30, 2004 (to allow assessment of lab values and medications in the last month of baseline) and November 30, 2008 (to allow for the 1-month exposure period and at least one day of follow-up). 

Measurements of TSAT were excluded if 1) iron dextran or both ferric gluconate and iron sucrose were administered during the exposure period; 2) fewer than 120 days of the 180-day baseline period were covered by Part A claims (which include dialysis claims), suggesting incomplete data; and 3) there were fewer than 9 dialysis sessions in the last month of baseline or the exposure period. We also excluded TSAT records with missing covariate information and TSAT measurements that occurred in the follow-up period of a prior eligible TSAT. The exposure assessment period began the day after the qualifying TSAT measurement.

### Study Variables

#### Outcomes

We examined four cardiovascular outcomes: death attributed to cardiovascular disease, hospitalization for myocardial infarction (MI), hospitalization for stroke, and a combined/composite outcome of any one of these three. These outcomes were determined by examining the CMS inpatient and outpatient claims and death notification data. The specific definitions for each outcome are presented in Table S1 in File S1. 

#### Exposures

The primary exposures of interest were: (1) high dose versus low dose iron administration and (2) bolus versus maintenance dosing. Based on the distribution of IV iron dosing, we defined high dose as > 200 mg of IV iron in the one month exposure period. Low dose was defined as 1-200 mg of IV iron. We also created a no iron category for individuals who did not receive any IV iron during the 1-month exposure period. For the 2-week and 1-week exposure periods, high doses were greater than 125 mg and 75 mg, respectively. 

A month was classified as a bolus month if it contained administrations of iron on consecutive dialysis sessions of at least 100 mg.  We also classified a month as a bolus month if it contained two or more administrations of iron > 100 mg that had the potential to exceed 600mg within 30 days based on spacing between the doses in the sequence.  For example, two consecutive iron doses of 200 mg each, within 10 days, would qualify as a bolus dose according to our definition. Months that had no bolus dosing patterns were classified as “maintenance months”. We also included a no iron category. 

#### Confounders

We included several covariates in our cohort analyses. Descriptions of the covariates and the manner in which they were defined in our models are presented in Table S2 in File S1. Covariates included demographic characteristics (e.g., age, sex, race, Medicaid eligibility, census region, year), clinical characteristics (e.g., cause of ESRD, vintage, body mass index (BMI), type of vascular access, number of hospital days), laboratory and anemia management variables (e.g. baseline hemoglobin, ferritin, TSAT, iron dose, EPO dose, albumin, receipt of a blood transfusion, EPO dose during exposure period), and several comorbidity measures based on the Elixhauser classification [15], and content expertise of the investigative team. Due to the extensive list of comorbidities, we selected ones to include in a parsimonious model and ones to add during sensitivity analyses.

### Statistical Analysis

To assess the relation between iron dosing practices and adverse outcomes, we used Cox proportional hazards regression analyses to estimate hazard ratios (HR) and a semiparametric additive risks model to estimate risk differences (RD), all accompanied by their respective 95% confidence intervals (CI) [16]. Individuals were censored at death (for the hospitalization outcomes), loss to follow-up, receipt of a kidney transplant, or administratively at the end of available data. We first estimated an unadjusted HR (e.g. high versus low dose) for each outcome and then a multivariable-adjusted HR that included age, sex, race, BMI, EPO dose during baseline and the exposure period, baseline hemoglobin, baseline ferritin, index TSAT, current use of a catheter vascular access, years on dialysis, serum albumin, number of days in hospital, any infection in last month, and history of the following in the last six months: diabetes, stroke, MI, pneumonia, vascular access infection, sepsis, chronic obstructive pulmonary disease, cancer, and gastrointestinal bleeding. 

We also conducted these analyses within several subgroups that were defined a priori based on demographic and clinical characteristics as defined in Table S3 in File S1. Individuals were categorized based on race, vintage, catheter use, history of infection in last month of baseline, TSAT levels at baseline, Ferritin levels at baseline, TSAT*Ferritin combinations (e.g., low TSAT and high ferritin) at baseline, albumin levels at baseline, and hemoglobin levels at baseline. 

#### Sensitivity Analyses

To assess the robustness of the results dependent on shorter exposure and/or follow-up periods, we repeated the analyses on other cohorts: i.e., two week exposure, six week follow-up; one week exposure, six week follow-up; one month exposure, six week follow-up. To assess the addition of other potentially relevant covariates, we conducted a sensitivity analysis where we added additional covariates: reported cause of ESRD, year, region, Medicaid eligibility, and a number of additional comorbidities (Table S2 in File S1). 

The UNC Institutional Review Board approved this study. The Institutional Review Board did not deem it necessary to obtain written consent for the use of de-identified patient information from the dialysis registry. 

## Results

A total of 117,050 patients met the study entry requirements and contributed 776,203 unique iron exposure/follow-up periods for comparisons of outcomes among dosing strategies, Figure 2. The patient characteristics of the primary cohort stratified by dosing pattern are presented in Table 1. The greatest number of exposure periods corresponded to our definition of maintenance iron dosing, followed by no iron (non-users), low dose, high dose, and bolus. The groups had generally similar demographic and medical characteristics with some notable exceptions. For example, the prevalence of dialysis catheters was higher in the bolus and high dose iron groups than in the maintenance or low dose groups. Mean hospital days in the past month were also higher for the bolus and high dose iron groups. The prevalence of comorbid conditions, both cardiovascular and other, was also higher in the bolus and high dose IV iron groups than in the other groups.

**Table 1:** Patient Characteristics at Baseline, by Exposure Group (N = 776,203).

**Characteristic**	**High (24.0%)**	**Low (37.8%)**	**Bolus (12.6%)**	**Maintenance (49.2%)**	**Non-user (38.2%)**
Mean (SD) Age, y	60.8 (15.0)	61.5 (15.0)	60.6 (15.1)	61.4 (14.9)	61.4 (14.9)
Female (%)	45.4	45.4	46.1	45.3	46.3
Race (%)					
White	49.2	49.1	47.5	49.6	47.9
Black	45.8	44.5	47.8	44.3	45.0
Medicaid (%)	50.9	51.0	51.9	50.7	51.6
Region (%)					
Midwest	18.0	16.8	17.4	17.2	16.2
Northeast	12.3	12.7	11.9	12.7	12.3
South	51.9	47.1	55.3	47.3	50.4
West	17.2	22.9	14.9	22.2	20.7
ESRD Reason (%)					
Diabetes	46.4	44.7	45.8	45.3	43.3
Glomerulonephritis	12.0	12.6	12.0	12.5	13.1
Hypertension	30.4	31.1	30.8	30.9	31.3
Mean (SD) Vintage, y	4.0 (4.1)	4.3 (4.4)	4.0 (4.1)	4.3 (4.3)	4.8 (4.6)
Mean (SD) BMI	27.4 (7.1)	27.3 (6.8)	27.1 (7.0)	27.4 (6.9)	26.6 (6.6)
Catheter (%)	23.8	21.8	25.5	21.8	20.4
Blood transfusion (%)	8.5	4.9	10.3	5.3	6.2
Hospital Days*, mean (sd)	0.8 (2.1)	0.5 (1.7)	1.0 (2.3)	0.5 (1.7)	0.5 (1.8)
Vascular access	13.4	9.7	15.0	10.1	8.9
Diabetes	56.6	52.1	57.1	53.0	50.9
Ischemic stroke	12.1	10.0	13.1	10.2	10.3
Myocardial infarction	4.2	2.9	4.6	3.1	3.1
COPD, Asthma	20.8	16.5	22.2	17.2	16.0
Cancer	9.2	8.1	9.8	8.2	8.4
Gasterointestinal bleeding	6.4	3.9	7.7	4.2	3.9
**Labs and Medications, mean (sd)**					
Index TSAT, %	24.7 (10.5)	30.4 (11.4)	22.4 (10.6)	29.7 (11.1)	35.0 (15.9)
Ferritin, mcg/L	489 (314)	536 (309)	481 (358)	527 (298)	766 (514)
Hemoglobin, g/dL	12.1 (1.4)	12.3 (1.2)	11.9 (1.5)	12.3 (1.2)	12.2 (1.3)
Albumin, g/dL	3.8 (0.4)	3.9 (0.4)	3.8 (0.4)	3.9 (0.4)	3.9 (0.4)
Baseline EPO, 1,000 units/mo.	112 (103)	78 (82)	127 (110)	82 (84)	70 (81)
Concurrent EPO, 1,000 units/mo.	107 (103)	76 (81)	123 (110)	79 (84)	74 (84)
Iron during baseline period, mg	331 (312)	189 (205)	354 (397)	216 (204)	89.0 (225.6)
Iron during exposure period, mg	538 (316)	135 (54)	689 (371)	190 (114)	0.0 (0.0)

ESRD= end stage renal disease, COPD= chronic obstructive pulmonary disease. * Hospital Days in previous month.

For the Bolus versus Maintenance groups, all variables were different at a significance level of p < 0.001, except for ESRD Reason (p=0.02).For High versus Low dose groups, all variables were different at a significance level of p< 0.00, except for frequency of female (p=0.97), and frequency of Medicaid (p=0.84).

Tables 2 shows the results for the high versus low dose comparison with respect to the cardiovascular outcomes. Compared to low dose, high dose IV iron was associated with an increased risk of cardiovascular events, including acute myocardial infarct and stroke, and cardiovascular death in unadjusted models. However, after adjustment for several covariates, there was no significant association, assessed either by HR, or RD. 

Comparison of bolus and maintenance dosing revealed similar associations to the high and low dose comparison. The unadjusted HR demonstrated an increased risk of all measured cardiovascular outcomes with bolus dosing, Table 3. With adjustment, however, the HR and RD were strongly attenuated towards the null. 

**Table 3:** Hazard Ratios and Risk Differences Comparing Bolus versus Maintenance Dosing (n=number of events).

**Parameter Estimate (95% CI)**	**Myocardial Infarction (n=6,078**)	**Stroke (n=8,618)**	**Cardiovascular Death (n=12,584)**	**Myocardial Infarction, Stroke, or Cardiovascular Death (n=25,350)**
**Unadjusted Hazard Ratio**	1.14 (1.06,1.23)	1.27 (1.19,1.35)	1.37 (1.30,1.44)	1.30 (1.25,1.34)
Adjusted* Hazard Ratio	0.98 (0.90,1.06)	1.05 (0.98,1.12)	1.02 (0.96,1.07)	1.03 (0.99,1.07)
Adjusted* Risk Difference (events per 1,000 person yrs.)	-0.82 (-3.9, 2.2)	2.5 (-1.6, 6.2)	0.90 (-3.2, 4.7)	3.7 (-2.4, 9.9)

* Adjusted analyses controlled for the following variables at baseline: age; race; sex; vintage; number of hospital days in last month; history of infection in last month; body mass index; most recent vascular access, hemoglobin; ferritin; index transferrin saturation; iron dose; albumin level; epoetin alfa dose; history in last 6 months of pneumonia, sepsis, vascular access infection, diabetes, stroke, myocardial infarction, chronic obstructive lung disease, cancer, gastrointestinal bleeding; and epoetin alfa dose during exposure. N=776,203

Results of the subgroup analyses are presented in Tables S4 and S5 in File S1 for the high versus low dose and bolus versus maintenance dosing comparisons, respectively. There were no consistent trends seen, though in the bolus versus maintenance comparison, individuals with high ferritin (500-800 mcg/L) and low TSAT (<25%) that were administered a bolus dose of iron had increased risks of cardiovascular death, and the composite outcome of death, myocardial infarction, or stroke. 

Sensitivity analyses using additional covariates, and with differing exposure and follow up periods, did not change the point estimates of the fully adjusted models as seen in either Table 2 or 3. These data are not shown. Furthermore, we observed no increase in risk of the outcomes under study among recipients of maintenance iron dosing compared to non-users of IV iron.

**Table 2:** Hazard Ratios and Risk Differences Comparing High Dose versus Low Dose (n=number of events).

**Parameter Estimate (95% CI)**	**Myocardial Infarction (n=6,078**)	**Stroke (n=8,618)**	**Cardiovascular Death (n=12,584)**	**Myocardial Infarction, Stroke, or Cardiovascular Death (n=25,350)**
**Unadjusted Hazard Ratio**	1.08 (1.01,1.15)	1.18 (1.12,1.25)	1.27 (1.22,1.33)	1.20 (1.16,1.24)
Adjusted* Hazard Ratio	0.94 (0.88,1.01)	1.01 (0.95,1.08)	1.01 (0.96,1.06)	0.99 (0.96,1.03)
Adjusted* Risk Difference (events per 1,000 person yrs.)	-2.4 (-4.8, 0.02)	0.42 (-2.7, 4.0)	0.05 (-3.4, 3.5)	-1.6 (-7.1, 3.9)

* Adjusted analyses controlled for the following variables at baseline: age; race; sex; vintage; number of hospital days in last month; history of infection in last month; body mass index; most recent vascular access, hemoglobin; ferritin; index transferrin saturation; iron dose; albumin level; epoetin alfa dose; history in last 6 months of pneumonia, sepsis, vascular access infection, diabetes, stroke, myocardial infarction, chronic obstructive pulmonary disease, cancer, gastrointestinal bleeding; and epoetin alfa dose during exposure. N=776,203

## Discussion

In this cohort study of a representative group of adult hemodialysis patients, we have examined the comparative safety of several IV iron supplementation strategies on short-term cardiovascular morbidity and mortality. To our knowledge, this is the largest and certainly most contemporary study to directly examine the comparative cardiovascular safety of common IV iron dosing strategies, which are used throughout many parts of the world, including the United States, Europe, and Japan [17,18]. Our data show that larger doses of intravenous iron are not associated with cardiovascular morbidity or mortality compared to relatively smaller doses. Furthermore, among the various pre-specified subgroups of patients, there were no consistent differences in cardiovascular events or mortality between patients receiving the various dosing strategies. 

There has been longstanding concern about the potential adverse cardiovascular effects of intravenous iron. Ferric iron (Fe^3+^) is reduced to ferrous iron (Fe^2+^) by leukocyte generated superoxide dismutase [19]. Ferrous iron leads to the generation of the reactive oxygen species, hydroxyl radical, known to damage membrane lipids, oxidize low-density lipoprotein, and promote atherogenesis [20]. It is believed that most intravenous iron formulations, including ferric gluconate and iron sucrose, release bioactive iron [8], especially if given rapidly enough to oversaturate receptors [21]. Indeed, studies with relatively small numbers of patients have suggested that intravenous iron use is associated with an important clinical pre-cursor of systemic atherosclerosis [22,23]; both studies, of 60 patients each, demonstrated a significant association of total intravenous iron exposure with carotid intimal media thickness.

Larger epidemiological studies however have not been able to translate the association of intravenous iron exposure with pre-clinical disease into more robust clinical outcomes. For example, Feldman and colleagues examined the effect of IV iron administration and mortality in a cohort of over 32,000 dialysis patients, and found no association of cumulative dose of IV iron with mortality [24]. A second study of over 58,000 patients by Kalantar-Zadeh and colleagues also did not find an association of iron dose with mortality, either all cause or cardiovascular mortality [25]. Rather, the administration of up to 400 mg was associated with improved survival overall, and among many relevant subgroups. At doses greater than 400 mg, there was a trend towards increased mortality. Our results complement and extend these previous studies. Like them, we did not find a consistent or meaningful association of IV iron with cardiovascular morbidity or mortality. The richness of the current data allowed us to examine clinically applicable dosing patterns, rather than merely cumulative doses. Furthermore, we were able to study specific outcomes related to cardiovascular morbidity, including hospitalization for stroke and myocardial infarction rather than just all-cause mortality. Finally, a particular strength of our study (and in contrast to previous studies), we had both patient-level electronic health records information from the dialysis provider and claims data from the public payer providing comprehensive and uniform insurance to these patients. 

The study should be interpreted in the context of the following limitations. First, since we focused on events during a 3-month period, examination of long-term effects of IV iron on cardiovascular events are not possible in this study. Yet, the focus on short-term safety minimizes common sources of bias in non-experimental studies with longitudinal exposures, including time-varying confounding, selection bias, and immortal person-time bias. This design improves the validity of the exposure being related to the outcome. Future analyses, perhaps using a marginal structural model [26], may be better suited to determine the long-term safety of IV iron.

Second, the study was non-experimental in design, and, therefore, could have been confounded by unobserved differences between patient groups. There was an increased prevalence of observed comorbidities among patients receiving either high or bolus dosing of iron. This would theoretically bias the association away from the null hypothesis and towards an observed association with the higher doses of IV iron. The rich clinical and administrative data found in the study allowed for adjustment for important clinical and laboratory variables, and in fact, there was no observed association of cardiovascular morbidity and mortality. 

The lack of an adverse cardiovascular safety signal should not be interpreted as an unmitigated endorsement for the use of large doses of IV iron. In an era of capitated payments for dialysis services, black box warnings for ESA use, and *dynamic* Quality Incentive Program performance measures [4,27,28] there may be strong incentives for the use of large doses of iron to manage anemia. Yet, another broad category of adverse events, infectious complications from the administration of intravenous iron, remains an unresolved issue that requires additional investigation [29-32]. Furthermore, a study on the effectiveness of larger doses of IV iron suggested only a marginal benefit on anemia parameters such as hemoglobin and EPO dose, compared to smaller doses of iron [33]. Thus, until additional evidence is available, the prudent choice may be moderation in the use of IV iron for the management of anemia in hemodialysis patients [34,35]. 

In summary, large doses of IV iron supplementation were not associated with short-term cardiovascular morbidity or mortality in a contemporary cohort of hemodialysis patients. However, additional safety concerns of IV iron administration, including potential infectious complications and long-term cardiovascular safety warrant further scrutiny. Ultimately, a controlled clinical trial comparing intravenous iron dosing strategies may be necessary to determine an approach that optimizes benefit and minimizes risk to patients. 

## Supporting Information

File S1 **Supporting information**.
Table S1, Study Outcomes Definitions and Data Source. Table S2, Definition of Covariates. Table S3, Definition of Subgroups. Table S4, Adjusted* Hazard Ratios (HR) and Risk Differences** (RD) for Other Adverse Outcomes, High Dose Versus Low Dose. Table S5, Adjusted* Hazard Ratios (HR) and Risk Differences** (RD) for Other Adverse Outcomes, Bolus versus Maintenance Dosing.
(DOCX)
