# Supplementary material for: Intravenous Iron Supplementation Practices and Short-Term Risk of Cardiovascular Events in Hemodialysis Patients
Source: PLoS One. 2013 Nov 1;8(11):e78930. doi: 10.1371/journal.pone.0078930 (PMC3815308; doi:10.1371/journal.pone.0078930)
Supplement: File S1 — Supporting information. Table S1, Study Outcomes Definitions and Data Source. Table S2, Definition of Covariates. Table S3, Definition of Subgroups. Table S4, Adjusted* Hazard Ratios (HR) and Risk Differences** (RD) for Other Adverse Outcomes, High Dose Versus Low Dose. Table S5, Adjusted* Hazard Ratios (HR) and Risk Differences** (RD) for Other Adverse Outcomes, Bolus versus Maintenance Dosing. (DOCX) [file pone.0078930.s001.docx]

**SUPPORTING INFORMATION S1**

**Table S1.**

| **Outcome** | **Definition** | **Data Source** |
| --- | --- | --- |
| CVD death | Primary cause of death: 23,26, 28, 29,36 | CMS death notification file |
| Hospitalized for MI | Primary ICD-9-CM code of 410.ss, hospital stay 3-180 days or died within 3 days | CMS Part A claims |
| Hospitalized for stroke | Any ICD-9-CM diagnostic code of 435.xx, 436.xx, 433.x1, 434.x1, 437.1x, 437.9x | CMS Part A claims |

**Table S2.**

| **COVARIATE** | **DEFINITION** | **SOURCE** |
| --- | --- | --- |
| ***Demographic*** | | |
| Age | Categorized as: 16-45; 46-60; 61-75; >75 yrs. | USRDS |
| Sex | Male or female | USRDS |
| Race | White, Black, Other | USRDS |
| Medicaid Eligibility | Indicator for dual eligibility during any part of the baseline | USRDS |
| Year of treatment | 2004, 2005, 2006, 2007, 2008 | Clinical Database |
| Census Region | Based on location of last dialysis center in baseline period: Northeast, South, Midwest, West | USRDS |
| ***Clinical*** | | |
| Vintage | Categorized as 0; 1-3; 4 or more yrs. | USRDS |
| ESRD Reason | Diabetes, Glomerulonephritis, hypertension, other | USRDS |
| BMI | Categorized as underweight, normal, overweight, obese | Clinical Database & USRDS |
| Access | Most recent vascular access (catheter vs fistula/graft) prior to TSAT index date | Clinical Database |
| ***Anemia Management*** | | |
| EPO dose (baseline) | Total EPO dose, (quintiles) | Clinical Database |
| EPO dose (exposure) | Total EPO dose, (tertiles plus a no-use category) | Clinical Database |
| Index TSAT | Last TSAT at baseline (quintiles) | Clinical Database |
| Iron dose | Total dose at last month of baseline, categorized as none, low (1-200 mg), or high (>200mg) | Clinical Database |
| Hemoglobin | Most proximal Hb lab prior to index TSAT date  (<10,10-11,>11-12,>12-13,>13) | Clinical Database |
| Ferritin | Most proximal serum ferritin prior to index TSAT date (quintiles) | Clinical Database |
| Albumin | At baseline (<3.3, 3.3-3.9, >3.9) | Clinical Database |
| ***Comorbidities*** | | |
| Hospital days in last month of baseline | Categorized as 0, 1-3, <=4 | USRDS, Medicare Part A Claims |
| Diabetes | Any ICD-9-CM diagnostic code of 250.xx in baseline period |  |
| Ischemic Stroke | Any ICD-9-CM diagnostic code of 434.01, 434.11, 434.91, 435, 436, 437, 438, V12.54 in baseline period |  |
| MI | Any ICD-9-CM diagnostic code of 410.xx in baseline period |  |
| COPD | Any ICD-9-CM diagnostic code of 490.xx-496.xx, 505.xx, 506.4 in baseline period |  |
| Cancer | Any ICD-9-CM diagnostic code of 173.3, 173.9, 174.0-175.9, 179-195, 196-199, 232.9, 233.0, 233.1, 300.29, 338.3, 789.51, 795.82, 799.4, V67.2, 200, 201, 202.0-202.3, 202.50-203.01,203.8, 238.6, 273.3 in baseline period |  |
| GI bleeding | Any ICD-9-CM diagnostic code of 578.xx in baseline period |  |
| ***Additional Comorbidities for Sensitivity Analyses*** | | |
| Pulmonary circulation disease | Any ICD-9-CM diagnostic code of 415.xx-417.xx in baseline period | USRDS, Medicare Part A & B Claims |
| Peptic Ulcer Disease | Any ICD-9-CM diagnostic code of 530.2, 531.xx-534.xx, V12.71 in baseline period |  |
| Liver disease | Any ICD-9-CM diagnostic code of 070.32, 070.33, 070.54, 456.0, 456.1, 456.20, 456.21, 571.0, 571.2, 571.3, 571.4, 571.5, 571.6, 571.8, 571.9, 572.3, 572.8, V42.7 in baseline period |  |
| Other neurological problem | Any ICD-9-CM diagnostic code 331.9, 332.0, 333.4, 333.5, 334-335, 340, 341, 345.0, 345.1, 345.4, 345.5, 345.8, 345.9, 348.1, 348.3, 780.3, 784.3 in baseline period |  |
| Substance abuse | Any ICD-9-CM diagnostic code 303.xx-305.xx in baseline period |  |
| Ischemic Heart disease, other heart disease, peripheral vascular disease, history of CABG, Stent, PTCA | Any ICD-9-CM diagnostic code of 411.xx-414.xx, 420.xx-429.xx, 785.o, V45.0, v53.3, 402.11, 402.91, 404.11, 404.12, 404.91, 404.93, 093.2, 746.3-746.6, v42.2, v43.3, v43.4441.xx-443.xx, 447.1, 557.1, 557.9, 444.xx-445.xx; Procedure codes of 00.66, 92982, 92985, 36.06, 36.07, 92980, 33510-33519 in baseline period |  |
| Hypertension | Any ICD-9-CM diagnostic code of 401.xx-405.xx, except 402.11, 402.91, 404.11, 404.13, 404.91, 404.93 in baseline period |  |
| Rheumatic heart disease | Any ICD-9-CM diagnostic code of 393.xx -398.xx in baseline period |  |
| Psychiatric problems | Any ICD-9-CM diagnostic code 295.xx-298.xx in baseline period |  |
| Autoimmune disorders | Any ICD-9-CM diagnostic code of 564.1, 696.0, 696.1, 695.4, 710.0, 701.0, 710, 714, 720, 725 in baseline period |  |
| Blood loss anemia | Any ICD-9-CM diagnostic code of 280.0 in baseline period |  |
| Transfusion | Indicator for receipt of one or more transfusions during the baseline period, based on HCPCS codes P9010, P9011, P9016, P9021, P9022, P9038, P9039, P9040, 36430 and ICD-9 codes 99.03, 99.04 |  |

**Table S3.**

| **Subgroup** | **Definition** | **Data Source** |
| --- | --- | --- |
| Race: Black / Non-Black | Categorized as Black race or White/Other race | USRDS |
| Vintage: < 1year /1-<4 yrs./ >4 yrs. | Years on dialysis, categorized into 3 groups | USRDS |
| Catheter | Most recent vascular access prior to index TSAT was catheter | Clinical Database |
| Recent Infection | Any hospital admission in the last month with one of the following ICD-9-CM diagnostic codes as the principal diagnostic code: 001–139, 254.1, 320–326, 331.81, 372–372.39, 373.0–373.2, 382–382.4, 383.0, 386.33, 386.35, 388.60, 390–393, 421–421.1, 422.0, 422.91–422.93, 460–466, 472–474.0, 475–476.1, 478.21–478.24, 478.29, 480–490, 491.1, 494, 510–511, 513.0, 518.6, 519.01, 522.5, 522.7, 527.3, 528.3, 540–542, 566–567.9, 569.5, 572–572.1, 573.1–573.3, 575–575.12, 590–590.9, 595–595.4, 597–597.89, 598, 599.0, 601–601.9, 604–604.9, 607.1, 607.2, 608.0, 608.4, 611.0, 614–616.1, 616.3–616.4, 616.8, 670, 680–686.9, 706.0, 711–711.9, 730–730.3, 730.8–730.9, 790.7–790.8, 996.60–996.69, 997.62, 998.5, and 999.3. Any claims with the following HCPCS codes for antibiotic use in last month of baseline: J3370, J0690, J0713, J0692, J0696, J1580, J3260, J0278, J1840, J1956. Any indication of IV antibiotic use in the last month of baseline. | USRDS, Medicare Part A Claims/ USRDS, Medicare Part A & B Claims/ Clinical Database |
| Hemoglobin (g/dL): <10/ 10-12/>12 | Most proximal hemoglobin lab, categorized into 3 groups | Clinical Database |
| Hypo-responsive to ESA | Baseline hemoglobin 11 g/dL or less, and EPO dose at baseline in the top quartile | Clinical Database |
| TSAT (%): <20/ 20 – <50/ 50 or more | Index TSAT (last TSAT of baseline period), categorized into 3 groups | Clinical Database |
| Ferritin (mcg/L): <200/ 200-500/ 501-1200/ >1200 | Ferritin measure most proximal to index TSAT, categorized into 4 groups | Clinical Database |
| TSAT(%) * Ferritin (mcg/L): <25 * <500/ <25 * 500-800/ <25 * >800/ >25% * <500/ >25% * 500-800/ >25% * >800 | 6 combinations based on index TSAT and Ferritin measured most proximal to index TSAT | Clinical Database |
| Albumin (g/dL): <3/ 3-<3.5/ 3.5-<3.8/ 3.8-<4/ >4 | Albumin level most proximal to index TSAT, categorized into 5 groups |  |
| Bolus Dosing | When the amount and spacing between consecutive iron doses during the exposure period created a pattern which, if continued, had the potential to yield a total dose exceeding 600mg within 30 days. | Clinical Database |
| Maintenance Dosing | Iron dosing during the exposure period not classified as bolus dosing. | Clinical Database |

**Table S4.**

| **Subgroup** | **Events** | **MI** | **Events** | **Stroke** | **Events** | **CVD Death** | **Events** | **MI/Stroke/CVD Death** |
| --- | --- | --- | --- | --- | --- | --- | --- | --- |
| Overall | 6,078 | -2.4 (-4.8, 0.02) | 8,618 | 0.42 (-2.7, 4.0) | 12,584 | 0.05 (-3.4, 3.5) | 25,350 | -1.6 (-7.1, 3.9) |
| Black | 2,216 | -1.2 (-5.0, 1.8) | 3,781 | -0.70 (-4.9, 3.9) | 4,513 | -1.0 (-5.0, 3.0) | 9,832 | -2.4 (-10.0, 5.9) |
| Non-black | 3,862 | -3.3 (-6.7, 1.3) | 4,837 | 1.5 (-2.2, 5.3) | 8,071 | 1.2 (-3.8, 7.0) | 15,518 | -0.56 (-7.7, 7.1) |
| Catheter | 1,570 | -2.9 (-8.5, 3.3) | 2,607 | 3.1 (-4.7, 12.6) | 3,998 | 2.1 (-5.8, 11.2) | 7,577 | 1.6 (-11.2, 12.6) |
| Drive (low TSAT, Hi Ferritin) | 1,303 | 4.8 (-1.2, 9.8) | 1,725 | -3.8 (-10.8, 2.4) | 2,730 | 7.6 (-0.82, 15.6) | 5,337 | 8.9 (-4.4, 21.6) |
| Recent infection | 753 | -10.9 (-17.2, -5.4) | 1,278 | 5.0 (-4.7, 14.3) | 2,245 | -4.0 (-15.6, 8.2) | 4,001 | -7.9 (-23.7, 9.7) |
| Hypo ESA (low Hgb, Hi EPO) | 420 | 11.6 (-0.82, 22.7) | 608 | -17.1 (-31.3, -4.2) | 1,338 | -8.7 (-28.9, 12.5) | 2,209 | -13.5 (-37.6, 8.9) |
| TSAT 0-20 | 1,251 | 1.9 (-3.5, 7.3) | 1,787 | -3.4 (-10.4, 4.0) | 3,037 | -2.6 (-11.9, 5.4) | 5,632 | -3.3 (-15.7, 7.7) |
| 21-50 | 4,465 | -2.2 (-5.3, 0.89) | 6,224 | 2.0 (-1.2, 5.4) | 8,776 | 1.3 (-2.8, 5.9) | 18,091 | 1.1 (-4.2, 7.2) |
| 50+ | 362 | -13.9 (-23.6, -4.5) | 607 | 1.2 (-12.0, 15.2) | 771 | -2.3 (-17.0, 11.3) | 1,627 | -13.4 (-36.0, 7.8) |
| Vintage <1 yr | 452 | -11.2 (-20.8, -0.60) | 628 | 8.2 (-3.4, 21.7) | 773 | -1.9 (-13.1, 11.0) | 1,723 | -3.6 (-22.1, 14.3) |
| 1-3 yr | 3,080 | -1.3 (-4.3, 2.6) | 4,473 | -0.32 (-4.7, 4.8) | 5,990 | 0.85 (-4.4, 5.6) | 12,558 | -0.18 (-6.6, 6.9) |
| 3+ yr | 2,546 | -2.2 (-5.9, 0.93) | 3,517 | -0.05 (-4.3, 4.2) | 5,821 | -0.69 (-5.5, 4.6) | 11,069 | -3.1 (-10.4, 3.5) |
| Albumin <3 | 169 | 8.7 (-5.8, 24.8) | 382 | -12.2 (-35.7, 18.6) | 1,154 | -37.0 (-82.3, 1.9) | 1,604 | -47.9 (-91.9, -3.1) |
| 3 - <3.5 | 920 | 0.41 (-8.5, 8.4) | 1,486 | -0.74 (-12.7, 11.0) | 2,801 | -2.9 (-17.0, 11.6) | 4,826 | -1.4 (-20.5, 16.5) |
| 3.5 - <3.8 | 1,586 | -0.76 (-7.0, 4.6) | 2,244 | 2.7 (-5.8, 9.9) | 3,228 | 2.8 (-4.5, 11.3) | 6,518 | 4.6 (-8.0, 17.5) |
| 3.8 - <4 | 1,374 | -2.6 (-7.0, 2.7) | 1,896 | 2.0 (-3.7, 7.7) | 2,350 | 2.7 (-3.5, 9.0) | 5,210 | 2.0 (-8.3, 11.9) |
| >=4 | 2,029 | -4.7 (-8.4, -1.4) | 2,610 | -0.49 (-4.6, 3.2) | 3,051 | 1.00 (-2.7, 5.3) | 7,192 | -3.5 (-9.7, 2.2) |
| Hemoglobin <10 | 291 | -7.5 (-20.1, 3.2) | 490 | -0.23 (-15.9, 20.5) | 926 | -19.0 (-41.4, 3.6) | 1,584 | -17.7 (-48.6, 10.4) |
| 10 - 12 | 2,522 | -1.6 (-5.7, 2.2) | 3,660 | -2.5 (-7.2, 2.6) | 5,425 | -2.4 (-8.9, 3.1) | 10,820 | -6.9 (-14.2, 1.8) |
| >12 | 3,265 | -2.6 (-6.3, 0.57) | 4,468 | 2.5 (-1.0, 6.1) | 6,233 | 3.5 (-1.4, 8.0) | 12,946 | 3.4 (-3.7, 9.4) |
| Ferritin <200 | 509 | -8.9 (-15.4, -2.3) | 795 | 6.0 (-2.0, 15.0) | 1,114 | -3.9 (-14.5, 5.9) | 2,252 | -7.2 (-23.3, 7.3) |
| 200 - 500 | 1,929 | -3.8 (-7.7, 0.47) | 2,755 | 3.3 (-1.5, 8.4) | 3,960 | -4.3 (-9.7, 1.8) | 8,037 | -4.1 (-11.6, 5.0) |
| 501 - 1200 | 3,170 | 1.0 (-3.2, 4.7) | 4,395 | -3.7 (-7.7, 0.85) | 6,322 | 4.6 (0.23, 9.5) | 12,886 | 2.2 (-6.0, 10.4) |
| >1200 | 407 | -10.1 (-26.9, 5.7) | 580 | 12.1 (-7.9, 30.4) | 1,024 | -7.5 (-31.0, 20.8) | 1,875 | -4.8 (-39.2, 30.8) |
| TSAT * Ferritin: <25 * <500 | 1,154 | -5.0 (-10.0, -0.57) | 1,645 | -0.31 (-6.0, 6.6) | 2,577 | -9.2 (-17.0, -0.96) | 4,979 | -13.0 (-23.1, -2.5) |
| <25 * 500 - 800 | 861 | 5.5 (-0.33, 11.7) | 1,144 | -3.4 (-10.1, 3.0) | 1,828 | 8.6 (-1.4, 18.5) | 3,551 | 8.4 (-6.3, 20.6) |
| <25 * >800 | 561 | 0.28 (-10.9, 12.7) | 751 | -1.2 (-17.3, 14.7) | 1,267 | -2.4 (-20.5, 14.9) | 2,397 | 6.6 (-17.8, 32.4) |
| >=25 * 500 - 800 | 1,163 | 2.3 (-3.5, 8.5) | 1,711 | -0.74 (-5.9, 4.9) | 2,255 | 5.2 (-2.5, 13.1) | 4,772 | 6.6 (-3.6, 15.9) |
| >=25 * <500 | 1,281 | -4.9 (-9.0, -0.72) | 1,897 | 9.4 (3.6, 14.9) | 2,487 | 0.40 (-4.5, 6.8) | 5,289 | 4.4 (-4.8, 13.8) |
| >=25 * >800 | 995 | -10.6 (-18.6, -0.70) | 1,377 | -3.2 (-16.0, 8.7) | 2,006 | 3.1 (-9.9, 16.3) | 4,062 | -9.0 (-26.5, 12.2) |

*Adjusted analyses controlled for the following variables at baseline: age; race; sex; vintage; number of hospital days in last month; history of infection in last month; body mass index; most recent vascular access, hemoglobin; ferritin; index transferrin saturation; iron dose; albumin level; epoetin alpha (EPO) dose; history in last 6 months of pneumonia, sepsis, vascular access infection, diabetes, stroke, myocardial infarction, chronic obstructive pulmonary disease, cancer, gastrointestinal bleeding; and EPO dose during exposure.

**Risk difference is the hazard difference per 1,000 person years.

**Table S5.**

| **Subgroup** | **Events** | **MI** | **Events** | **Stroke** | **Events** | **CVD Death** | **Events** | **MI/Stroke/CVD Death** |
| --- | --- | --- | --- | --- | --- | --- | --- | --- |
| Overall | 6,078 | -0.82 (-3.9, 2.2) | 8,618 | 2.5 (-1.6, 6.2) | 12,584 | 0.90 (-3.2, 4.7) | 25,350 | 3.7 (-2.4, 9.9) |
| Black | 2,216 | -3.9 (-7.7, -0.18) | 3,781 | 2.2 (-3.3, 7.7) | 4,513 | 0.55 (-6.0, 6.1) | 9,832 | 0.13 (-8.7, 8.3) |
| Non-black | 3,862 | 2.1 (-2.2, 6.8) | 4,837 | 2.9 (-2.0, 8.3) | 8,071 | 2.1 (-4.1, 9.1) | 15,518 | 8.1 (-0.68, 18.3) |
| Catheter | 1,570 | 1.4 (-4.5, 8.2) | 2,607 | 0.04 (-6.5, 9.4) | 3,998 | 2.8 (-6.4, 14.4) | 7,577 | 4.8 (-7.6, 19.3) |
| Low TSAT, High Ferritin) | 1,303 | 0.96 (-5.3, 6.6) | 1,725 | 2.0 (-4.9, 9.1) | 2,730 | 9.4 (1.2, 17.7) | 5,337 | 13.3 (1.3, 27.2) |
| Recent infection | 753 | -9.8 (-18.6, -3.0) | 1,278 | 2.3 (-10.0, 13.9) | 2,245 | -0.83 (-13.0, 14.0) | 4,001 | -4.8 (-23.9, 13.3) |
| Hypo ESA (low Hgb, Hi EPO) | 420 | 9.3 (-0.38, 19.3) | 608 | -12.5 (-24.8, 0.92) | 1,338 | -8.8 (-28.1, 11.8) | 2,209 | -8.7 (-33.1, 18.0) |
| TSAT 0-20 | 1,251 | 3.9 (-1.7, 9.3) | 1,787 | 1.9 (-4.8, 8.4) | 3,037 | -1.3 (-8.5, 7.5) | 5,632 | 4.9 (-5.0, 16.1) |
| 21-50 | 4,465 | -1.4 (-5.0, 2.9) | 6,224 | 5.3 (0.71, 9.9) | 8,776 | 3.1 (-2.2, 8.6) | 18,091 | 8.2 (1.7, 17.2) |
| 50+ | 362 | 0.44 (-12.2, 15.1) | 607 | -14.3 (-29.6, 2.5) | 771 | 0.13 (-20.2, 22.3) | 1,627 | -10.6 (-39.4, 19.5) |
| Vintage <1 yr | 452 | -8.8 (-19.2, 3.0) | 628 | 6.2 (-7.1, 19.9) | 773 | 8.4 (-5.5, 22.4) | 1,723 | 8.4 (-13.2, 29.6) |
| 1-3 yr | 3,080 | -0.02 (-3.8, 4.7) | 4,473 | 2.3 (-2.5, 7.6) | 5,990 | -2.1 (-8.7, 4.2) | 12,558 | 3.0 (-7.4, 13.2) |
| 3+ yr | 2,546 | -0.16 (-5.1, 4.4) | 3,517 | 2.1 (-3.1, 7.9) | 5,821 | 3.0 (-3.2, 9.8) | 11,069 | 3.7 (-6.1, 12.3) |
| Albumin <3 | 169 | -2.7 (-21.9, 15.1) | 382 | -17.8 (-43.9, 10.7) | 1,154 | -27.3 (-66.9, 11.8) | 1,604 | -41.4 (-92.6, 2.0) |
| 3 - <3.5 | 920 | -0.69 (-8.4, 8.7) | 1,486 | 10.7 (-2.1, 23.6) | 2,801 | 14.5 (-2.5, 30.8) | 4,826 | 22.9 (1.2, 44.6) |
| 3.5 - <3.8 | 1,586 | -0.32 (-7.7, 6.4) | 2,244 | 1.8 (-7.6, 9.4) | 3,228 | -0.48 (-11.2, 9.1) | 6,518 | 4.8 (-9.2, 17.9) |
| 3.8 - <4 | 1,374 | 0.58 (-5.8, 7.7) | 1,896 | 1.7 (-6.3, 9.0) | 2,350 | -4.1 (-12.7, 4.6) | 5,210 | -1.9 (-15.2, 9.7) |
| >=4 | 2,029 | -1.7 (-6.0, 2.8) | 2,610 | 2.4 (-3.2, 7.2) | 3,051 | 2.6 (-2.2, 8.5) | 7,192 | 3.9 (-4.7, 12.6) |
| Hemoglobin <10 | 291 | 0.33 (-9.8, 10.9) | 490 | -2.0 (-14.8, 17.6) | 926 | 9.4 (-1.8, 18.5) | 1,584 | -24.5 (-49.1, 11.0) |
| 10 - 12 | 2,522 | -1.1 (-6.7, 4.0) | 3,660 | 3.3 (-2.1, 9.3) | 5,425 | 0.05 (-3.5, 3.7) | 10,820 | 3.1 (-7.0, 13.3) |
| >12 | 3,265 | -0.75 (-5.1, 3.4) | 4,468 | 2.4 (-3.1, 6.6) | 6,233 | -1.9 (-5.4, 1.7) | 12,946 | 7.5 (-0.92, 16.5) |
| Ferritin <200 | 509 | -6.3 (-12.2, -0.25) | 795 | 1.4 (-7.8, 10.7) | 1,114 | -4.1 (-16.3, 5.5) | 2,252 | -6.6 (-22.1, 8.4) |
| 200 - 500 | 1,929 | 0.36 (-4.7, 5.5) | 2,755 | 3.3 (-2.9, 9.6) | 3,960 | -0.06 (-7.3, 8.5) | 8,037 | 4.0 (-5.5, 15.1) |
| 501 - 1200 | 3,170 | 0.39 (-5.1, 5.1) | 4,395 | 2.8 (-3.0, 9.3) | 6,322 | 3.3 (-3.5, 11.6) | 12,886 | 7.5 (-2.1, 17.8) |
| >1200 | 407 | 0.22 (-16.1, 21.6) | 580 | -1.8 (-23.1, 17.6) | 1,024 | 11.0 (-17.4, 41.9) | 1,875 | 16.3 (-18.0, 65.7) |
| TSAT * Ferritin: <25 * <500 | 1,154 | -1.1 (-6.4, 4.4) | 1,645 | 2.7 (-4.3, 8.3) | 2,577 | -4.2 (-12.5, 3.3) | 4,979 | -2.3 (-13.1, 8.2) |
| <25 * 500 - 800 | 861 | 1.7 (-5.9, 9.4) | 1,144 | 3.4 (-4.2, 12.6) | 1,828 | 11.3 (0.75, 22.6) | 3,551 | 14.2 (0.24, 29.6) |
| <25 * >800 | 561 | -2.0 (-14.4, 9.6) | 751 | -2.6 (-19.9, 14.0) | 1,267 | -2.2 (-21.7, 18.1) | 2,397 | 6.4 (-23.8, 32.4) |
| >=25 * 500 - 800 | 1,163 | 5.1 (-3.4, 15.1) | 1,711 | 7.5 (-4.9, 17.7) | 2,255 | -3.2 (-16.2, 10.9) | 4,772 | 10.2 (-8.0, 27.6) |
| >=25 * <500 | 1,281 | -3.2 (-8.5, 3.2) | 1,897 | 4.2 (-2.8, 13.6) | 2,487 | 1.0 (-8.6, 10.9) | 5,289 | 4.5 (-7.7, 18.7) |
| >=25 * >800 | 995 | 1.6 (-10.5, 14.6) | 1,377 | 7.0 (-10.4, 23.6) | 2,006 | 14.6 (-5.4, 33.7) | 4,062 | 24.9 (-2.6, 52.4) |

*Adjusted analyses controlled for the following variables at baseline: age; race; sex; vintage; number of hospital days in last month; history of infection in last month; body mass index; most recent vascular access, hemoglobin; ferritin; index transferrin saturation; iron dose; albumin level; epoetin alpha (EPO) dose; history in last 6 months of pneumonia, sepsis, vascular access infection, diabetes, stroke, myocardial infarction, chronic obstructive pulmonary disease, cancer, gastrointestinal bleeding; and EPO dose during exposure.

**Risk difference is the hazard difference per 1,000 person years.
